# An Energy-Efficient Dynamic Feedback Image Signal Processor for Three-Dimensional Time-of-Flight Sensors

**DOI:** 10.3390/s24216918

**Published:** 2024-10-28

**Authors:** Yongsoo Kim, Jaehyeon So, Chanwook Hwang, Wencan Cheng, Jong Hwan Ko

**Affiliations:** 1 Department of Semiconductor and Display Engineering, Sungkyunkwan University, Suwon 16419, Republic of Korea; yongsookim@g.skku.edu; 2 Samsung Electronics, Hwaseong 18448, Republic of Korea; 3 Department of Electrical and Computer Engineering, Sungkyunkwan University, Suwon 16419, Republic of Korea; judenause@g.skku.edu (J.S.); ghkdcks12@g.skku.edu (C.H.); 4 Department of Computer Science, National University of Singapore, Singapore 117417, Singapore; wccheng@nus.edu.sg

**Keywords:** image signal processor, ToF sensor, dynamic feedback, energy efficiency, 3D depth image, point cloud

## Abstract

With the recent prominence of artificial intelligence (AI) technology, various research outcomes and applications in the field of image recognition and processing utilizing AI have been continuously emerging. In particular, the domain of object recognition using 3D time-of-flight (ToF) sensors has been actively researched, often in conjunction with augmented reality (AR) and virtual reality (VR). However, for more precise analysis, high-quality images are required, necessitating significantly larger parameters and computations. These requirements can pose challenges, especially in developing AR and VR technologies for low-power portable devices. Therefore, we propose a dynamic feedback configuration image signal processor (ISP) for 3D ToF sensors. The ISP achieves both accuracy and energy efficiency through dynamic feedback. The proposed ISP employs dynamic area extraction to perform computations and post-processing only for pixels within the valid area used by the application in each frame. Additionally, it uses dynamic resolution to determine and apply the appropriate resolution for each frame. This approach enhances energy efficiency by avoiding the processing of all sensor data while maintaining or surpassing accuracy levels. Furthermore, These functionalities are designed for hardware-efficient implementation, improving processing speed and minimizing power consumption. The results show a maximum performance of 178 fps and a high energy efficiency of up to 123.15 fps/W. When connected to the hand pose estimation (HPE) accelerator, it demonstrates an average mean squared error (MSE) of 10.03 mm, surpassing the baseline ISP value of 20.25 mm. Therefore, the proposed ISP can be effectively utilized in low-power, small form-factor devices.

## 1. Introduction

In recent years, various cutting edge technologies and devices based on artificial intelligence (AI) have been developed. This has led to increased attention towards image and video information processing techniques for their utilization. Among these, object recognition and detection are key research topics in the field of computer vision. The primary objective is to accurately identify objects within images and videos [1,2]. In this domain, various types of sensor can be employed, and time-of-flight (ToF) sensors have emerged as one of the most widely used sensor types in recent years [3,4]. ToF sensors are generally more precise in providing accurate 3D geometric data and work well in a variety of ambient lighting situations compared to other methods [5]. These characteristics have raised interest in the field of 3D image technology using 3D ToF sensors, leading to various research efforts [6,7]. Recently, these sensors have been integrated into small devices. They are being applied in various research areas such as human pose estimation, hand pose estimation (HPE), and facial recognition. As a result, there is an increasing demand for ToF sensors that prioritize energy efficiency, as well as accuracy and reliability [8,9,10,11]. However, 3D ToF sensors generate large amounts of raw data. The hardware required to process these complex 3D images in real time demands significant computational resources and high power consumption. Furthermore, the domains that primarily utilize ToF sensors, such as augmented reality (AR), virtual reality (VR), and various biometric recognition fields, are predominantly deployed on low-power, small form-factor devices, such as mobile and edge devices [12]. Therefore, optimizing resource utilization in 3D image processing is of paramount importance to enhance energy efficiency in these devices.

In previous research, the focus was primarily on improving the analog circuits of 3D ToF sensors, with limited attention given to the digital circuits responsible for post-processing, such as the image signal processor (ISP) [13,14,15]. However, with the increasing complexity of 3D video processing tasks and the growing volume of 3D data, there has been a heightened interest in efficient data management and post-processing. Generally, processes or algorithms for data processing are complex, making implementation with analog circuits difficult and often inefficient. For example, the process of calculating depth using phase signals from a ToF sensor and converting the results into a point-cloud format requires complex computations. This makes it very difficult and inefficient to implement with analog circuits. Therefore, by implementing these functions through a digital circuit-based ISP and integrating it with the sensor, it is possible to provide various and efficient post-processing techniques and algorithm solutions based on flexible design methods, thereby improving the quality and efficiency of 3D image processing [16,17,18,19].

In this paper, we propose an ISP that supports a dynamic feedback configuration to maximize the performance of 3D ToF sensors and enhance energy efficiency. In applications using 3D ToF sensors, it is common to receive various forms of unpredictable 3D images depending on the surrounding environment. In such situations, conventional ISPs can degrade the performance of the application by receiving and processing unnecessary object information from unwanted areas and delivering it to the application. In addition, when using a sensor that operates at a fixed resolution, the ISP must perform numerous calculations (e.g., depth computation and point-cloud conversion) for every pixel in each frame, even when high-resolution images are not required. These situations ultimately increase system latency and power consumption, reducing energy efficiency.

The proposed dynamic feedback ISP has a wide range of practical applications, especially in fields where efficient real-time processing and energy-saving capabilities are crucial. For instance, AR and VR devices, such as smart glasses, can greatly benefit from the ISP’s ability to process only the necessary regions with optimal resolution, thereby extending battery life. Additionally, autonomous vehicles and robotics rely on ToF sensors for navigation and object detection. In such scenarios, our ISP’s dynamic resolution adjustment ensures that only the relevant areas are processed in high resolution, enhancing reaction speed and reducing latency. Moreover, medical applications, including hand gesture recognition for surgical assistance, demand precise and fast real-time processing. The proposed ISP can optimize performance by dynamically focusing on relevant areas, thereby ensuring low latency and high accuracy. Similarly, security and surveillance systems can utilize this ISP to operate continuously in power-constrained environments. For example, only areas with detected motion can be processed in high resolution, while other regions remain idle, leading to significant energy savings.

These practical use cases demonstrate that the proposed ISP is well-suited for modern applications requiring low power consumption, high performance, and precise processing. The ability to dynamically adjust resolution and extract valid areas in real time not only reduces computational overhead but also ensures that the system can adapt to changing environments, making it highly applicable to a variety of edge devices.

The overall contributions of this paper can be summarized as follows:

(1) We propose a dynamic area extraction technique that continuously extracts the required valid area from sensor images based on real-time feedback from the application. This reduces computation for unnecessary areas, thereby increasing energy efficiency, reducing processing time, and providing more reliable data to the application.
(2) We introduce a dynamic resolution technique that adjusts resolution based on feedback from the application and the previous frame’s valid object information, using pixel grouping for logical resolution changes. This efficiently manages processing time, power consumption, and resources according to system operating conditions.
(3) We propose an ISP that efficiently manages resources by using optimized pipeline design and operational control methods, maximizing energy efficiency and minimizing power consumption.

The rest of this paper is organized as follows. In Section 2, we introduce and review research related to energy-efficient ISPs. In Section 3, we provide a detailed explanation of the techniques applied to the dynamic feedback ISP proposed in this paper. Then, in Section 4, we introduce an application system that combines an energy-efficient dynamic feedback image signal processor with a real-time hand pose estimation accelerator. The experimental results of the proposed ISP are discussed in Section 5, and finally, the paper is concluded in Section 6.

## 2. Related Work

Technologies such as AR, VR, and autonomous driving are rapidly evolving and becoming more prevalent in daily life. As a result, there has been an increase in the use of high-resolution, high-frame-rate images based on 3D depth image sensors. This substantially increases the volume of pixel data that must be processed by sensors and ISPs, resulting in higher power consumption and greater resource use. However, 3D depth image sensors are primarily used in mobile and edge devices, which typically have limited resources. As a result, addressing these challenges is crucial. Consequently, various studies are underway to mitigate these issues and efficiently process image information.

Recently, studies have been published that implement depth information processing capabilities on hardware such as FPGAs to directly interface with 3D depth image sensors in order to improve performance and efficiency. F. Chen [16] notes the increasing use of 3D time-of-flight (ToF) sensors and the corresponding shortage of 3D image signal processors (ISPs) capable of supporting these sensors. And they also introduce the implementation of an energy-efficient real-time ISP for 3D ToF sensors on an FPGA. J. Seiter [20] has also developed a depth calculator system for 3D ToF sensors on FPGA, which utilizes minimal resources and power consumption. N. Druml [21] proposes a platform that processes raw data from 3D ToF sensors to calculate depth using hardware implemented on an FPGA, which is then integrated into an environment that can leverage software for diverse data transformations to improve usability. These prior studies have focused on efficiently implementing features for processing 3D depth images on performance-enhancing hardware.

However, enhancing performance through hardware implementation has its limitations, and eventually, additional functionalities may be necessary for better performance. Among these additional features, research into identifying regions of interest (ROIs) or utilizing them in future frames is actively ongoing. These studies improve energy efficiency and reduce operating time by identifying the ROI and only activating or post-processing the pixels within this area. M. Casares et al. [22] reduced energy consumption and processing time by utilizing tracking information from previous frames. This information was used to identify ROI areas in subsequent frames and process only those areas. Additionally, the camera was switched to an idle state based on the speed of changes or movements in the foreground objects. Iqbal et al. [23] verified the performance of an FPGA-based implementation by adopting an adaptive subsampling algorithm to improve the energy efficiency of embedded vision systems. Although this algorithm is limited to a single ROI, it predicts the location of the ROI in future frames and turns off the pixels outside this area, enabling vision applications to utilize the ROI programming feature.

In addition, recent developments have introduced multitasking and multimodality techniques into ISPs, making them highly useful for handling various tasks. Yu et al. [24,25] proposed a multitasking model using infrared thermal imaging to simultaneously perform identity recognition and contact time estimation, demonstrating potential applications in crime investigation and security. Nguyen et al. [26] developed a few-shot learning-based multitasking model for detecting and segmenting camouflaged animals, achieving strong performance, even with limited data. Similarly, the ISP we propose performs multitasking by applying both the dynamic area extraction technique and the dynamic resolution technique. This approach enables efficient processing of both tasks simultaneously.

Such studies have explored various additional functionalities to enhance sensor operation efficiency using ROI techniques. However, implementing these additional ROI detection/tracking features requires more resources, which can lead to increased power consumption and operational time. Thus, implementing hardware with minimal overhead for additional performance-enhancing features can offer significant benefits. In particular, if the hardware can dynamically process and provide the optimal image form required by an application in real-time through feedback, it allows for efficient hardware configuration and operation. In addition, since the application already detects or tracks the ROI to perform its task, efficient hardware configuration can be achieved by utilizing this information as feedback without adding additional functionality. This paper proposes an image signal processor (ISP) for 3D depth image sensors that utilizes feedback information in the form of point clouds provided by the application to dynamically adjust the area or resolution of images while minimizing the implementation of additional features. Through this approach, we demonstrate that implementing all functions on an FPGA optimized for hardware operation using minimal resources can reduce operational time and enhance energy efficiency.

## 3. Method

In this section, we introduce the dynamic area extraction technique and the dynamic resolution technique proposed in this paper. The dynamic area extraction technique identifies the area containing valid objects based on feedback information received from the application. The dynamic resolution technique dynamically adjusts the resolution of the valid area based on feedback information from the application. This approach allows the ISP to process only the information corresponding to the ROI in the entire input image based on the application’s feedback and optimally adjust the resolution for that area. Consequently, the ISP can process images efficiently, and the application can receive the necessary information more accurately.

**Figure 1:**
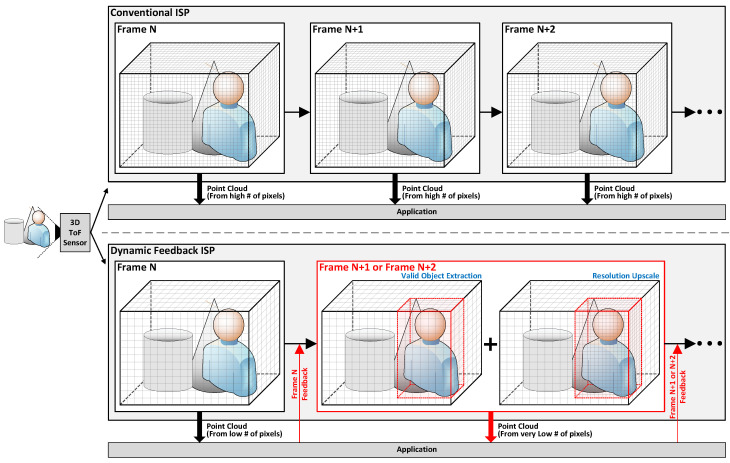
A general overview of the operation comparison between a conventional ISP and the proposed dynamic feedback ISP. The proposed dynamic feedback ISP can reduce the number of pixels used compared to the conventional ISP, resulting in reduced computation and power consumption, thus enhancing energy efficiency. (#: Number).

As seen in the example of the conventional ISP in Figure 1, typically, the raw image obtained from the sensor may include objects unrelated to the target object, or the ratio of the valid object in the entire image may be low, complicating the identification of the target object. Therefore, this paper proposes a method to extract valid areas along the X, Y, and Z axes from the raw 3D images of each frame based on feedback received from the application in order to minimize computations on unnecessary areas.

Additionally, with a fixed resolution, accurately identifying valid objects becomes difficult when they are mixed with unnecessary ones, as it is impossible to increase the resolution for more precision. Conversely, when valid objects are already clearly identified and a lower resolution would suffice, a fixed resolution can lead to unnecessarily high resolution and reduced operational efficiency. Therefore, as shown in the example of the dynamic feedback ISP in Figure 1, a method is proposed to dynamically adjust the resolution through pixel grouping based on the application’s feedback. This approach ensures optimal image quality suitable for the application’s operation in each frame.

**Figure 2:**
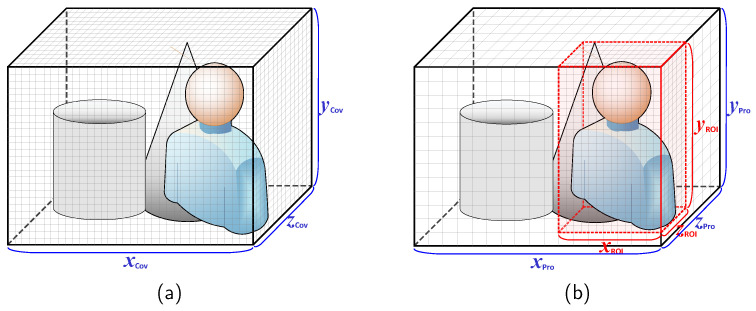
Comparison of pixel usage between the conventional method and the proposed method. (**a**) Example of conventional method. (**b**) Example of proposed method.

The effectiveness of the proposed approach can be illustrated with the following example. As shown in Figure 2a, the traditional method processes 1280, 960, and 400 pixels along the $x_{Cov}$, $y_{Cov}$, and $z_{Cov}$ axes, respectively, requiring the same amount of pixel data to be processed for every frame. However, as demonstrated in Figure 2b, with the method proposed in this paper, the first frame can be processed with reduced resolution, such as 320, 240, and 100 along the $x_{Pro}$, $y_{Pro}$, and $z_{Pro}$ axes, enabling rough processing. For subsequent frames, the application feedback can be used to identify ROI information, and only the ROI can be processed at a higher resolution, such as 640, 480, and 200 along the $x_{\mathrm{ROI}}$, $y_{\mathrm{ROI}}$, and $z_{\mathrm{ROI}}$ axes. This approach enables more efficient processing of the ROI compared to the traditional method. This example is provided for illustrative purposes, and the efficiency may vary dynamically depending on different environments and conditions. Equation (1) presents a formula that calculates the efficiency ratio based on the resolution levels used.

(1) $$\frac{x_{Cov}}{x_{\mathrm{ROI}}}\times \frac{y_{Cov}}{y_{\mathrm{ROI}}}\times \frac{z_{Cov}}{z_{\mathrm{ROI}}}=Efficiency$$

Additionally, the proposed method can help improve the performance of applications compared to traditional methods. For the hand pose estimation application presented as an example system in this paper, it can enhance the accuracy of hand pose estimation. This is because, instead of randomly sampling a fixed number of points from the entire image, the proposed ISP extracts points solely from the ROI. As a result, the number of valid points corresponding to meaningful objects increases, which directly improves the accuracy of the application. Equation (4) demonstrates the improvement in accuracy achieved by the proposed method shown in Equation (3) compared to the traditional method described in Equation (2).

(2) $$N_{\mathrm{valid}}=\frac{A_{\mathrm{valid}}}{A_{\mathrm{total}}}\times N_{\mathrm{total}}$$

(3) $$N_{\mathrm{valid}}^{\mathrm{ROI}}=N_{\mathrm{ROI}}\;\left(A_{\mathrm{valid}}\subseteq A_{\mathrm{ROI}}\right)$$

(4) $$\frac{N_{\mathrm{valid}}^{\mathrm{ROI}}}{N_{\mathrm{valid}}}>1$$

**Notations:**

- $N_{\mathrm{valid}}$: The number of points containing valid objects
- $A_{\mathrm{valid}}$: The area containing valid objects
- $A_{\mathrm{total}}$: The total area of the image
- $N_{\mathrm{total}}$: The number of points sampled from the entire image
- $N_{\mathrm{valid}}^{\mathrm{ROI}}$: The number of valid points within the ROI
- $N_{\mathrm{ROI}}$: The number of points sampled from the ROI

In the proposed method, the accuracy of the application is improved by focusing on extracting valid points from the ROI instead of sampling from the entire image. In conventional approaches, a fixed number of points ($N_{\mathrm{total}}$) are sampled from the entire image, which often includes a large portion of irrelevant or background areas. As a result, the number of valid points corresponding to meaningful objects ($N_{\mathrm{valid}}$) is limited by the ratio of the valid area to the total image area, $\frac{A_{\mathrm{valid}}}{A_{\mathrm{total}}}$. However, in the proposed ISP, the sampling is restricted to the ROI, ensuring that the points correspond more accurately to the objects of interest. Since the ROI is selected to encompass the valid area $\left(A_{\mathrm{valid}}\subseteq A_{\mathrm{ROI}}\right)$, the number of valid points in the ROI sampling ($N_{\mathrm{valid}}^{\mathrm{ROI}}$) increases compared to the conventional approach.

This increase in valid points enhances the accuracy of the application, as it allows the system to focus its computational resources on the meaningful portions of the data, rather than on irrelevant areas. The effectiveness of the proposed method can be quantified with Equation (4), indicating that the ROI-based sampling results in a higher proportion of relevant points, thereby improving the overall accuracy and performance of the application.

**Figure 3:**
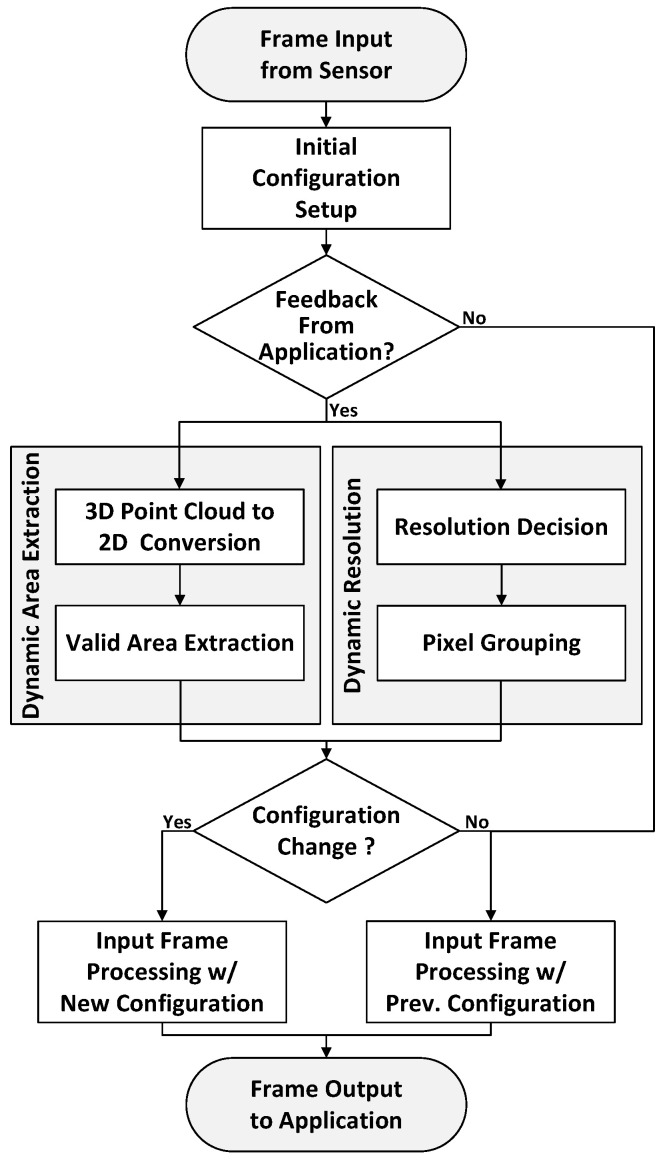
Overall flow chart for dynamic feedback operation.

Through these proposed methods, the application can obtain more suitable preprocessed 3D images rather than using raw 3D images, thereby reducing unnecessary tasks and computations, decreasing power consumption, and enabling more efficient operation. Figure 3 presents the overall operation flow of the proposed feedback operation in a flowchart.

### 3.1. Dynamic Area Extraction Technique

3D images from sensors usually contain unprocessed raw data, which often lack suitability for direct use by applications. For example, the raw 3D image received from the sensor may include background or surrounding elements that are not of interest to the application, which could interfere with the functional operations, such as inference performed by the application, potentially leading to accuracy losses and ultimately affecting the overall performance. Additionally, the additional computations required for the unnecessary parts can cause increased operation time and power consumption.

**Figure 4:**
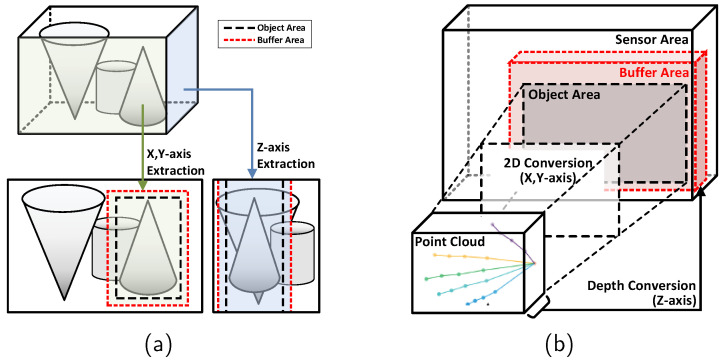
Dynamic area extraction technique. (**a**) Example of valid area extraction. (**b**) Valid area extraction mechanism.

Therefore, we propose a dynamic area extraction technique to identify core areas required by the application and make them available for utilization within the application. Figure 4a shows an example of applying valid area extraction to a 3D image. Applying this method to ISPs allows the processing of raw data only for the areas used in the actual application. The ISP receives feedback in the form of point-cloud information representing the actual and valid areas used by the application. Based on this feedback, only the actual valid areas from the entire data of the next frame input from the ToF sensor is computed and processed, and then passed to the application. The processing of this feedback data is carried out in two stages, as described below. The first method is the 3D point cloud to 2D conversion method. It converts 3D point-cloud feedback into 2D information, mapping it to the sensor’s input image to identify the valid area used by the application. The second method is the valid area extraction method. This method uses the valid area information obtained from the first method to extract and process only the data in that area. Algorithm 1 explains the detailed operation algorithm of the dynamic area extraction technique.

#### 3.1.1. 3D Point Cloud to 2D Conversion

Our work utilizes an application that provides feedback on areas of interest in the form of a 3D point cloud. This feedback is generated based on the application’s own task-specific criteria and depth information from the previous frame produced by the ToF sensor. We then interpret and map these areas as ’valid’ regions using our proposed method for further processing. However, the majority of sensors output raw images in RGB-D format, rather than providing actual 3D coordinates. Therefore, it is essential to process these 2D raw images to accurately identify and localize valid objects. To achieve this, we must first reverse transform the 3D point-cloud data of the valid areas, received as feedback from the application, into 2D pixel information. This information is then mapped onto the raw 2D image and incorporated into the subsequent input frame. To generate bounding boxes through the processing of these subsequent input frames, we have employed several algorithms used for coordinate transformations, which are explained below.

**Algorithm 1** Dynamic Area Extraction Technique  1:**Initialize:** curr_area = init_value  2:   3:**while** (Dynamic_Area_Extraction_En == 1) **do**  4:      **if** (user_reset == 1) **then**  5:            next_area = init_value  6:      **else if** (b_box <= curr_area) **then**  7:            next_area = b_box  8:            up_res_flag = 1  9:      **else if** (b_box > curr_area) **then**10:            **if** ((b_box - curr_area) > limit) **then**11:               next_area = b_box12:               down_res_flag = 113:          **else**14:               next_area = b_box15:               up_res_flag = 116:          **end if**17:      **else**18:            next_area = curr_area19:      **end if**20:      curr_area = next_area21:**end while****Abbreviations:**-init_value: Initial area configured by user-curr_area: Current area-b_box: Bounding box area-limit: Threshold value for area change-down_res_flag: Flag to decrease resolution-up_res_flag: Flag to increase resolution

To convert a 3D point cloud into 2D pixel coordinates, it is essential to understand the transformation of depth data from a 2D depth image into a 3D point cloud, as shown in Algorithm 2 [27]. Thisalgorithm leverages transformation processes to map depth data to spatial coordinates. The algorithm incorporates critical camera parameters, such as the principal point coordinates ($c_{x},c_{y}$), focal lengths along the *x* and *y* axes ($f_{x},f_{y}$), and the dimensions of the depth image (height and width). These parameters facilitate the geometric transformations required to convert pixel-based depth values into their corresponding 3D coordinates. The algorithm iterates through each pixel in the depth image, extracts the depth value, and computes the 3D coordinates (x,y,z) by adjusting the pixel positions based on the principal point and scaling by the focal lengths.
**Algorithm 2** Depth to point clouds**Require:** Depth_image, $c_{x},c_{y},f_{x},f_{y},height,width$**Ensure:** x,y,z  1:**for** *i* in range height **do**  2:    **for** *j* in range width **do**  3:        z= depth_image[i][j]  4:        $x=\left(j-c_{x}\right)*z/f_{x}$  5:        $y=\left(i-c_{y}\right)*z/f_{y}$  6:    **end for**  7:**end for**

We used Algorithm 2 to derive Algorithm 3, which projects 3D coordinates back onto the 2D image plane. Using projection equations, it calculates the 2D pixel coordinates ($px_{x},px_{y}$) by scaling the 3D coordinates with the focal lengths and adjusting for the principal point. This process effectively translates the 3D spatial location back to its corresponding position in the 2D image. Through this algorithm, we obtain the coordinates of pixels used in the valid area extractor via pipelining.
**Algorithm 3** Point clouds to 2D pixel coordinates**Require:** $x,y,z,c_{x},c_{y},f_{x},f_{y}$**Ensure:** $px_{x},px_{y}$  1:$px_{x}=\left(x*f_{x}/z\right)+c_{x}$  2:$px_{y}=\left(y*f_{y}/z\right)+c_{y}$

#### 3.1.2. Valid Area Extraction

The valid area extraction method defines bounding boxes for extracting valid areas in order to accurately determine the area where objects exist in 3D images. Figure 4b illustrates how the valid area extraction method works. It uses the 2D positions of valid objects from the previous frame, obtained through the previously described process of converting point clouds into 2D, to determine the valid pixel areas in the X and Y directions in the 3D image of the next frame. Simultaneously, it determines the valid range in the Z direction based on the depth information of valid objects from the previous frame. At this time, to account for potential changes in object size, movement, and environmental conditions between frames, users are allowed to define the valid object area by adding a certain margin level around the area received as feedback from the application in each direction.

The prevalent approach to accomplish the valid area extraction is to project the 3D point-cloud representations onto the 2D image plane, and subsequently delineate the boundaries by performing coordinate-wise comparisons between the projected 2D pixel coordinates. However, the method of converting all 3D point clouds into pixels involves numerous calculations and comparisons, which can result in latency and increased power consumption. Therefore, to address the computational complexity and inefficiency associated with this conversion process, we propose a simple approximation method based on the maximum and minimum values of the coordinates *x* and *y* of the point cloud. To begin the method, the minimum and maximum values of both *x* and *y* coordinates within the point cloud must be determined. This process yields four ordered pairs: $\left\{Min\left(x\right),Z_{min(x)}\right\},\left\{Max\left(x\right),Z_{max(x)}\right\},\left\{Min\left(y\right),Z_{min(y)}\right\}$, and $\left\{Max\left(y\right),Z_{max(y)}\right\}$. Here, Min(x) and Max(x) represent the minimum and maximum values of the *x* coordinates in the point cloud, respectively, with $Z_{min(x)}$ and $Z_{max(x)}$ being the corresponding *z* coordinate values at these *x* coordinates. The *y* coordinates follow similarly, with Min(y) and Max(y) denoting the minimum and maximum values, and $Z_{min(y)}$ and $Z_{max(y)}$ as the corresponding *z* coordinate values.

**Figure 5:**
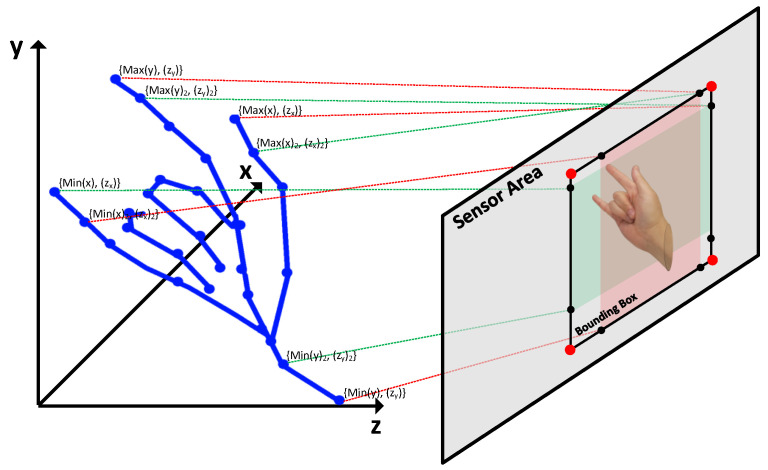
Example of bounding box decision.

Figure 5 is an example of projecting a 3D point-cloud area onto a sensor-based 2D plane. In the figure, the blue lines represent the 3D points, and their projections on the sensor plane form the bounding box indicated by red dots. This bounding box accurately captures the spatial extent of the point cloud in the 2D image, compensating for the depth-based discrepancies in the pixel coordinates.

However, considering Algorithm 3, which calculates pixel coordinates based on depth, it becomes evident that the maximum and minimum values of the point cloud may not align with those of the pixel coordinates. For instance, consider two points, A(3,$y_{A}$,4) and B(2,$y_{B}$,2), within the point cloud, where the camera parameters are $f_{x}=2$ and $c_{x}=4$. When applying Algorithm 3, we obtain pixel coordinates $px_{x}\left(A\right)=5$ and $px_{x}\left(B\right)=6$. Despite $x_{A}>x_{B}$ in the point cloud, the pixel coordinate $px_{x}\left(A\right)<px_{x}\left(B\right)$. To address this discrepancy, we extend our consideration to the second-largest and second-smallest alignment pairs. This results in eight ordered pairs: $\left\{Min\left(x\right),Z_{min(x)}\right\},\left\{Max\left(x\right),Z_{max(x)}\right\},$$\left\{Min\left(y\right),Z_{min(y)}\right\}$, $\left\{Max\left(y\right),Z_{max(y)}\right\}$, and their respective second-largest and second-smallest pairs. This allows us to define a reasonably accurate bounding box with just eight transformation and comparison operations, resulting in advantages in terms of latency and power consumption.

### 3.2. Dynamic Resolution Technique

The resolution of images captured through the depth sensor is determined by the number of pixels supported by the sensor. By utilizing all available pixels from the sensor, we can obtain more precise depth image data by using the maximum resolution supported by the sensor. However, this approach increases the amount of raw data that the ISP needs to process, resulting in higher computational demands and higher power consumption. Therefore, this paper proposes a dynamic resolution technique that allows for logical resolution control depending on the situation.

The dynamic resolution technique uses a pixel grouping method that consolidates multiple pixels into a single pixel or utilizes each pixel individually. This method adjusts the resolution of the next frame higher or lower based on the application’s feedback information for the current frame and the results of the dynamic area extraction technique, supporting the efficient operation of the application. Algorithm 4 explains the detailed operation algorithm of the dynamic resolution technique.
**Algorithm 4** Dynamic Resolution Technique  1:**Initialize:** c_p_group = init_value  2:   3:**while** (Dynamic_Resolution_En == 1) **do**  4:      **if** (user_reset == 1) **then**  5:            n_p_group = init_value  6:      **else if** (up_res_flag == 1) **then**  7:            **if** (c_p_group == 1) **then**  8:                 n_p_group = c_p_group  9:            **else**10:                 n_p_group = c_p_group - user_def11:            **end if**12:      **else if** (down_res_flag == 1) **then**13:            **if** (c_p_group == 6) **then**14:                 n_p_group = c_p_group15:            **else**16:                 n_p_group = c_p_group + user_def17:            **end if**18:      **else**19:            n_p_group = c_p_group20:      **end if**21:      c_p_group = n_p_group22:**end while****Abbreviations:**-init_value: Initial pixel group configured by user-c_p_group: Current pixel group-n_p_group: Next pixel group-user_def: User define value for pixel group change-down_res_flag: Flag to decrease resolution-up_res_flag: Flag to increase resolution

#### 3.2.1. Resolution Decision

**Figure 6:**
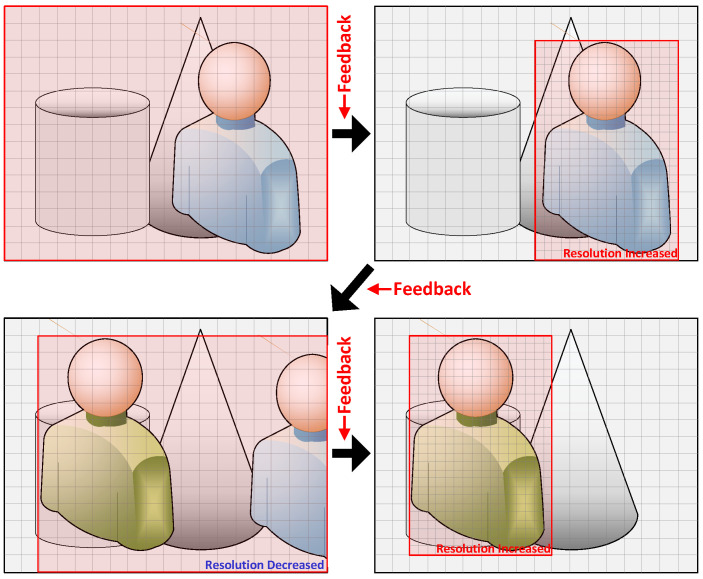
Example of resolution decision. Red box: valid area.

The resolution decision method derives the resolution to be applied to the next frame based on the feedback information from the application. The ISP detects areas containing valid objects in each frame received from the sensor and adjusts the resolution based on the application’s feedback. Figure 6 illustrates an example of the resolution decision.

At the initial operation, the ISP starts with a low resolution, delivering rough, low-resolution overall image information to the application. Based on this, the application performs its initial operations and generates information about the actual necessary valid area within the overall image, providing this feedback to the ISP. When processing the next frame, the ISP increases the resolution only for the extracted valid area based on this feedback, allowing the application to receive more precise valid area images. By increasing the resolution only for the valid area instead of the entire image, unnecessary computations are reduced, while the application can generate the needed images more accurately, thus processing images much more efficiently.

Conversely, if the valid area information is reset by the user’s settings or if the position or size of the valid area changes beyond the user-defined threshold, the system will lower the resolution to the initial state. This indicates that the previously set valid area has significantly changed, suggesting a substantial alteration in the target or surrounding environment. Consequently, the system repeats the aforementioned process to accurately identify the valid area and adjust the resolution accordingly. Through this mechanism, the ISP operates at the optimal resolution depending on the situation, maximizing efficiency.

#### 3.2.2. Pixel Grouping

As the resolution of the input image increases, the amount of computation required to process that image also increases. If the resolution of the image could be adjusted flexibly according to the situation, the amount of data to be processed would change accordingly, enabling efficient operation. However, if the sensor itself does not have real-time resolution control functionality, changing the resolution in real time is not easy. Therefore, a method to adjust the image resolution in real time without modifying the existing sensor would be very useful. Therefore, we propose a pixel grouping method that logically groups a fixed number of pixels supported by the sensor to effectively adjust the resolution of the entire image or specific areas. This grouping method provides the flexibility to adjust the resolution to an appropriate level depending on the content of the images received by the sensor.

Resolution adjustment is implemented through the pixel grouping method based on the results of the resolution decision method described above. Table 1 shows the example of pixel grouping approach integrated into the ISP proposed in this paper. As seen in Algorithm 4, based on the feedback information and image characteristics, one of the four pixel grouping methods (representative pixel data, maximum pixel data, minimum pixel data, or average pixel data) is selected, and the system adjusts the settings in the direction of the selected pixel group using the user_def value. The ISP then logically groups the pixels according to this setting or the user’s choice to adjust the resolution. For example, when grouping four pixels in a 2 × 2 configuration from a ToF sensor that supports a maximum resolution of 1280 × 960, selecting one representative pixel can reduce the overall image resolution to a maximum of 640 × 480. At this point, the number of pixels selected in each group is fixed, but the specific positions of the selected pixels are determined based on the user’s settings. This approach significantly reduces the pixel information that the ISP needs to process, lowering the computational load, reducing power consumption, and conserving resources while greatly improving the processing speed.

## 4. Proposed System Architecture

**Figure 7:**
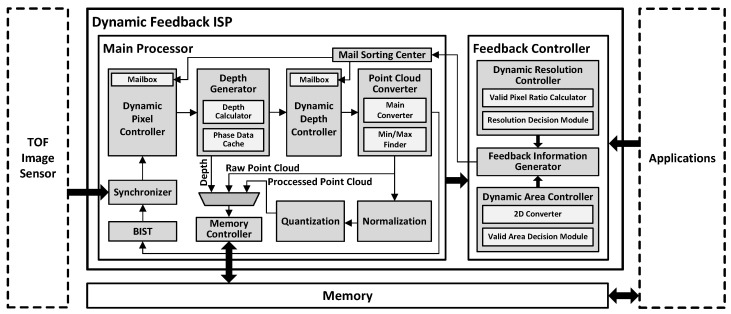
Overall block diagram of proposed example hardware based on a dynamic feedback ISP.

In this section, we propose an example hardware architecture of an application system based on the dynamic feedback ISP. The proposed ISP is designed to operate at optimal performance when utilizing feedback information from the application. Therefore, we have developed a system that uses the 3D ISP for a hand pose estimation accelerator application. Figure 7 illustrates the block diagram of the entire example system. The dynamic area controller extracts the valid areas in each frame based on feedback from the application. Then, the dynamic pixel controller and dynamic depth controller extract pixel data from these valid areas for use in the application. Additionally, the dynamic resolution controller analyzes the quality level of the images needed for each frame, and based on this analysis, the dynamic pixel controller can dynamically adjusts the resolution for the next frame by controlling the pixel information. In other words, all feedback information is comprehensively analyzed and evaluated by the feedback controller, then applied to the next frame by the main controller for use in the application.

### 4.1. Main Processor

The main processor plays a crucial role in processing the raw data received from the ToF sensor into a suitable format for use in applications. It consists of a dynamic pixel controller that processes pixel data received from the sensor based on feedback information, a depth generator for computing depth information, a dynamic depth controller for selective depth direction filtering based on feedback information, a point-cloud converter for transforming depth information into point-cloud format, and a quantization and normalization module for post-processing point-cloud data.

**Figure 8:**
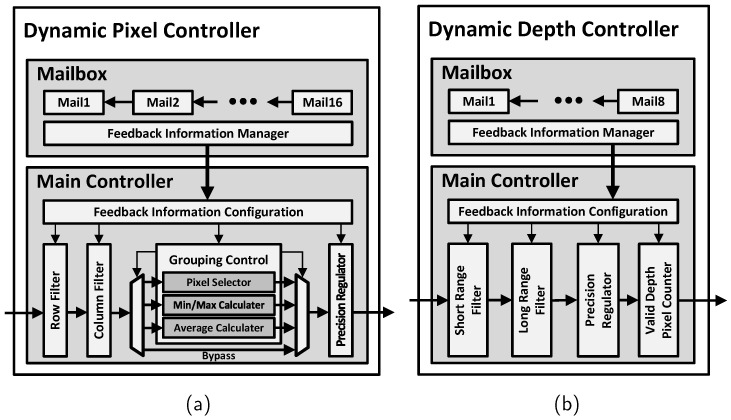
Hardware details in main processor. (**a**) Dynamic pixel controller. (**b**) Dynamic depth controller.

The dynamic pixel controller can finely control raw pixel data from ToF sensors to extract data from valid areas or adjust the resolution as needed. Figure 8a illustrates the details of the dynamic pixel controller, and Table 2 provides a summary of the functions. Based on the information received from the feedback controller, row and column filters are used to selectively pass only the information of pixels within the valid area of the incoming frame to the next stage. The selected pixels are grouped by the grouping controller based on the resolution information obtained from the feedback. Depending on the configuration, the minimum, maximum, or average values of the grouped pixels are computed, followed by precision adjustment before output. All these operations are designed to be processed serially and continuously as soon as they are inputted from the sensor, ensuring that there is no delay in real-time operation.

The dynamic depth controller is responsible for extracting valid areas in the depth direction. Figure 8b illustrates the details of the dynamic depth controller, and Table 2 provides a summary of the functions. First, the pixel information transmitted from the previous step is selectively filtered using short-range and long-range filters to retain only those pixels that fall within the valid depth range. Through this process, information such as out-of-focus sensor data or unnecessary background can be effectively filtered. These filtered pixels are then transformed by the point-cloud converter and transmitted to the application. This allows the application to perform computations only on the pixels that belong to the required areas, thereby enhancing the efficiency of the hardware operation. Simultaneously, the valid depth pixel counter measures the number of pixels within the valid depth range, providing auxiliary information for resolution determination to the dynamic resolution controller.

The feedback information received from the application may arrive at irregular intervals, depending on the type of application and its operating environment. Therefore, the mailboxes within the dynamic pixel controller and dynamic depth controller are designed to continuously receive and manage feedback delivered irregularly from the application via the feedback controller. Additionally, they verify the validity of the feedback information and control its sequential processing through scheduling.

### 4.2. Feedback Controller

The feedback controller receives feedback information from the application, processes it into a suitable format for feedback operation, and generates the configuration information that is applied for the next frame. The feedback controller consists of a dynamic area controller, a dynamic resolution controller, and a feedback information generator.

The dynamic area controller generates information for valid area extraction based on feedback information. This enables the main processor to extract core areas more accurately for delivery to the application. The 2D converter translates the 3D valid area information received as feedback from the application into 2D information. The valid area decision module utilizes this converted information to match it with the sensor’s pixel areas and generate information about the valid areas used by the application from the input data received from the sensor. The size, shape, or position of objects in the valid area may vary across input frames depending on the environmental conditions. Taking these factors into account, the system determines the expanded final valid area by additionally reflecting the margin information input by the user.

The dynamic resolution controller generates information to dynamically adjust the resolution of each frame based on the quality of the 3D images obtained from the ToF sensor and feedback information from the application. The valid pixel ratio calculator calculates the ratio of valid depth pixels computed by the dynamic depth calculator and forwards this ratio to the resolution decision module.

The resolution decision module evaluates the quality of the 3D image based on the depth information analyzed by the valid pixel ratio calculator using the user-input valid pixel threshold and determines the resolution for the next frame.

The feedback information generated by the dynamic area controller and dynamic resolution controller is consolidated through the feedback information generator. This integrated feedback information is transmitted to the main processor, where it is utilized in processing the pixels received from the ToF sensor.

### 4.3. Pipelined Architecture

**Figure 9:**
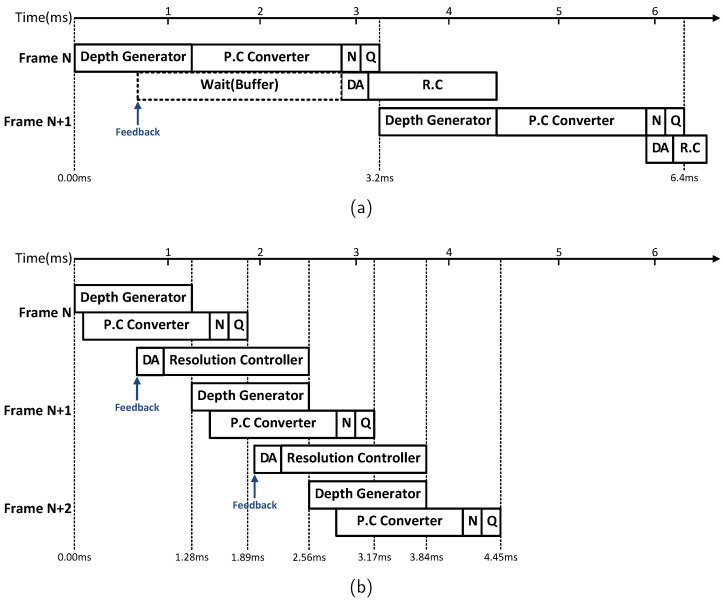
Comparison of sequential and pipelined operation flow. (**a**) Example of sequential operation flow. (**b**) Example of pipelined operation flow. P.C: Point Cloud, N: Normalization, Q: Quantization, DA: Dynamic area controller, R.C: Resolution controller.

The proposed ISP employs pipeline architecture throughout its operation to enhance hardware efficiency. We modularized the functions required for ISP operation to align with the pipeline structure, then arranged these modules to optimize performance in hardware. Figure 9 illustrates the operation of the proposed ISP when processing a 640 × 480 resolution image, comparing the sequential architecture with the pipeline architecture. In the sequential structure, each function is executed step-by-step, resulting in increased processing time proportional to the number of frames. In contrast, the pipeline architecture is structured to maximize module utilization, making its benefits more pronounced as the number of frames increases. This approach achieves approximately 2.48× higher performance when processing the same number of frames compared to the sequential structure. Additionally, each module is designed to operate only when required, with power shut off during idle states, optimizing power consumption by carefully managing the timing of each pipeline stage.

## 5. Experimental Results

### 5.1. Implementation Results

The proposed ISP with dynamic feedback was implemented on an FPGA to assess its performance and efficiency. The baseline ISP processes all the pixel data from the 3D ToF sensor for each frame according to the conventional method and delivers them to the application. On the other hand, the proposed ISP optimizes and delivers each frame’s data to the application using the dynamic feedback technique.

The proposed ISP was synthesized on the Xilinx Kintex UltraScale KU115 FPGA evaluation platform using Xilinx Vivado 2022.2, with a clock frequency of 100 MHz. Table 3 shows the resource utilization and performance of the baseline ISP and the proposed ISP with the applied technique. The proposed ISP, compared to the baseline ISP, showed an increase of approximately 39% in LUT usage, approximately 34% in FF usage, and an additional 2 DSPs. The increase in resources was a result of adding a feedback controller, dynamic pixel controller, and dynamic depth controller to the proposed ISP. Despite this increase, the efficiency of the proposed ISP improved by approximately 6.4×. Additionally, power consumption was reduced by 10%, indicating a significant performance enhancement compared to the additional resources used.

### 5.2. Performance Evaluation

#### 5.2.1. Performance Evaluation of Hand Pose Estimation

A sample system, using a hand pose estimation application, was set up to evaluate the proposed ISP’s performance. For this purpose, we implemented a hand pose estimation accelerator based on the recently introduced HandFoldingNet [28] model to construct the example system. This setup allowed us to assess the performance of the proposed ISP and analyze its impact on the performance improvement of the application using it. The proposed sample system operates by post-processing the 3D image data input from the ToF sensor in the ISP. This processed data is then used by the accelerator to estimate hand poses. However, since 3D images directly captured using the ToF sensor do not provide ground truth for evaluating the results of hand pose estimation, quantitative analysis and evaluation are not feasible.

Therefore, we used the NYU dataset [29] to quantitatively analyze and evaluate the impact of the functional differences between the proposed ISP with dynamic feedback features and the baseline ISP on the final performance of the hand pose estimation accelerator. The NYU dataset includes hand pose information for evaluating the performance of various models and algorithms. It uses continuous video-format images to observe feedback changes frame-by-frame. It also contains other surrounding backgrounds along with hand poses, making it suitable for evaluating the performance of the proposed ISP. However, since the NYU dataset is provided in RGBD format, it cannot be directly used with an ISP that uses phase information as input. Therefore, we directly input depth information into the ISP’s point-cloud converter to analyze and evaluate the performance of the hand pose estimation accelerator. Additionally, since the NYU dataset shows little variation in hand shape, movement, and environmental changes, the margin applied to the valid area was set to the system default of 10%, and the resolution decision threshold was set to the system default of 85%. The proposed example system is implemented on an FPGA to perform hand pose estimation.

Table 4 shows the results of performing the hand pose estimation accelerator based on the baseline ISP and the proposed ISP using the original images from the NYU dataset. Essentially, all original images contain not only the hand but also people and background objects. When using the baseline ISP for such images, as seen in the results, all information in the image, including the background, is transmitted to the hand pose estimation accelerator. Additionally, because the area occupied by the hand is very small due to the unnecessary background, the accelerator has to distinguish this and perform hand pose estimation, resulting in higher errors and lower accuracy. As seen in Table 4, the unnecessary background information is removed by feedback and only actual hand information is transmitted in the proposed ISP. Therefore, the hand pose estimation errors are decreased, and more accurate results are achieved. Consequently, the baseline ISP showed an average distance error of 20.25 mm, while the proposed ISP showed an average distance error of 10.03 mm, indicating a reduction of more than 50%.

#### 5.2.2. Hardware Performance Evaluation

The proposed ISP demonstrates superior processing speed and lower power consumption compared to the baseline ISP, resulting in higher energy efficiency. As shown in Table 3, the baseline ISP supports a maximum resolution of 1280 × 960 but operates at a fixed resolution at all times, whereas the proposed ISP enables dynamic resolution adjustment, ranging from 1280 × 960 to 320 × 240. Therefore, while the baseline ISP achieves a maximum frame rate of 31 fps at a clock frequency of 100 MHz, the proposed ISP achieves a faster frame rate of approximately 178 fps as the resolution and image size dynamically change depending on the situation. Additionally, when performing the hand pose estimation application based on the NYU dataset used in this paper, it achieved a power consumption of 1.45 W and a high energy efficiency of 123.15 fps/W. Furthermore, the proposed ISP arbitrarily selects only 1024 pixels to convert to a point cloud and delivers this to the application to reduce the computational load required to convert all incoming pixels to a point cloud. This reduction is possible because the proposed ISP operates with the valid area and optimal resolution through feedback.

We also conducted a comparative analysis with previous research. It was challenging to find directly related studies controlling ISP operation based on feedback from 3D ToF sensors. However, we have compiled similar related research in Table 5. M. Yoshimura et al. [19] proposed a dynamic ISP similar to our study, where the parameters for each frame can be controlled based on the recognition results of the previous frame. They experimented using a GPU with software implementation using RGB sensor input data. They evaluated five parameters: auto gain (AG), denoiser (DN), sharpener (SN), gamma tone mapping (GM), and contrast stretcher (CS). Their results indicate that their proposed approach outperforms the others. However, these functions are all implemented in software and run on a GPU, which significantly limits their practicality and energy efficiency when used as an ISP that needs to operate closely with the sensor. F. Chen et al. [16], Z. Chen et al. [30], and I. Gyongy et al. [31] proposed ISPs based on 3D ToF sensors with additional features and implemented them in hardware. In summary, these studies focused solely on implementing ISP functions and improving their performance. They did not propose any special features, such as utilizing feedback, as we have in our study. Compared to these approaches, our proposed method not only provides fundamental ISP functions but also incorporates application feedback. This addition leads to lower power consumption, superior performance, and higher energy efficiency, demonstrating the advantages of our approach. Furthermore, this allows efficient hardware configuration and operation without compromising the performance of the application.

## 6. Conclusions

In this paper, we proposed an ISP specific to 3D ToF sensors that can optimize their functionality by dynamically changing settings based on feedback from the application. The proposed ISP is fully compatible with applications that can provide point-cloud feedback. It has been validated for hardware implementation and performance on the Xilinx KU115 FPGA evaluation board. The first technique that we introduce is the dynamic area extraction technique, which uses feedback information from the application. It calculates and post-processes only the valid areas necessary for the application to operate for each frame of input from the sensor. This approach reduces unnecessary computations while utilizing valid area information, thereby maintaining accuracy and increasing operational efficiency. The second proposed technique is the dynamic resolution technique, which determines the resolution based on changes in the valid area due to the dynamic area extraction technique or feedback from the application. It evaluates valid pixels by considering feedback and valid area information and then adjusts the resolution based on those results. These two techniques work together in a complementary manner. They allow the application to operate efficiently by improving performance, preserving accuracy, reducing power consumption, and decreasing operation time. Furthermore, when implemented in the FPGA, we apply the pipeline architecture and a low-power scheme to achieve even more efficient operation. In the results, we confirm that the proposed method supports achieving an accuracy equal to or greater than that of the conventional approach for the application. Additionally, we observed outstanding energy efficiency, reaching 123.15 fps/W.

In conclusion, the proposed dynamic feedback ISP not only improves energy efficiency and processing speed but also shows great potential for real-world applications such as AR/VR devices, autonomous vehicles, medical systems, and security solutions. By enabling efficient operation with minimal resources, the ISP is well-suited for use in edge devices that require both high performance and low power consumption.

Nonetheless, the proposed ISP is optimized for ToF sensors and is currently designed to handle a single ROI using only point-cloud feedback, which presents certain limitations. In future research, we plan to enhance and extend the feedback functionality to process multiple ROIs simultaneously and to accommodate various types of feedback information. Additionally, we aim to expand the ISP’s capabilities to support a wider range of sensors, improve compatibility, enhance performance, and optimize power consumption.

## Figures and Tables

**Table 1:** Example of pixel grouping based on representative pixel selection.

	Grouping	Valid Pixel	Resolution	Computational Ratio
1	Base Line	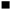	1280 × 960	100%
2	4 Pixels Grouping	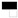	1280 × 480	50%
3		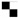	640 × 960	50%
4		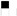	640 × 480	25%
5	16 Pixels Grouping	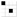	320 × 480	12.5%
6		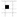	320 × 240	6.25%

**Table 2:** Summary of the functions of the modules within the dynamic pixel controller and dynamic depth controller.

Module	Function	Functionality
Common	Mailbox	Manage and schedule the feedback received from the application.
Dynamic pixel controller	Row filter	Filter pixels in the sensor’s row direction based on feedback.
	Column filter	Filter pixels in the sensor’s column direction based on feedback.
	Grouping control	Group pixels and perform calculations such as min/max/average on the selected pixels.
	Precision regulator	Adjust the precision of the final calculation results to be usable by the ISP.
Dynamic depth controller	Short-range filter	Filter pixels that fall outside the valid depth range in the direction close to the sensor.
	Long-range filter	Filter pixels that fall outside the valid depth range in the direction far from the sensor.
	Precision regulator	Adjust the depth precision of the selected pixels to be usable by the ISP.
	Valid depth pixel counter	Count the number of pixels that remain after the filtering process.

**Table 3:** Resource utilization and performance comparison with baseline ISP.

	Implementation				Performance				
	LUT	FF	DSP	BRAM	Resolution	Frame Rate (fps)	Power (W)	Efficiency (fps/W)	Error ^a^
Baseline	17,736	23,036	0	4	1280 × 960 (fixed)	31 (@100 Mhz)	1.61	19.25	20.25 mm
Proposed	24,657	30,975	2	4	1280 × 960 (variable) ^b^	178 (@100 Mhz)	1.45	123.15	10.03 mm

^a^ This is measured using the HandFoldingNet model application with NYU datasets. ^b^ The resolution changes dynamically, as proposed in this paper.

**Table 4:** Hand pose estimation results of baseline and proposed system based on HandFoldingNet model on NYU dataset.

Original	Baseline	Proposed	Original	Baseline	Proposed
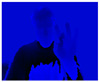	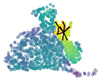	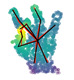	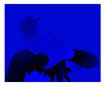	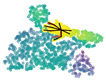	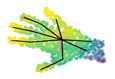
	Error = 7.09 mm	Error = 4.39 mm		Error = 8.89 mm	Error = 7.44 mm
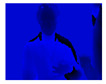	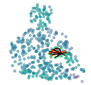	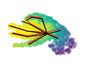	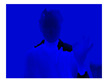	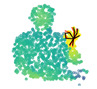	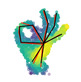
	Error = 39.07 mm	Error = 12.3 mm		Error = 14.15 mm	Error = 7.86 mm
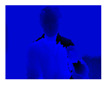	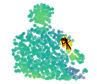	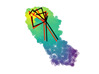	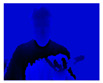	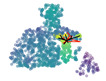	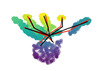
	Error = 22.16 mm	Error = 10.06 mm		Error = 22.10 mm	Error = 4.39 mm

**Table 5:** Performance comparison with related work.

Work	Sensor Type	Implementation	Performance	Major Components
[19]	RGB	Software (GPU:RTX2080)	Latency: 27.7 ms, 36 fps, 49.6% (AP@0.5:0.95) ^a^	Latent update style ISP controller, End-to-end optimization method
[30]	3D ToF	FPGA	256 × 256, 90 fps (50 Mhz), Error: 1 mm	Depth calculation(CORDIC algorithm), Gaussian filter
[16]	3D ToF	FPGA (XCZU9EG) 21,002 LUTs, 20,032 FFs, 39 BRAMs, 19 DSPs	1280 × 960, 60 fps (100 Mhz), 2.1W, Precision: 0.09 mm	Depth and intensity caculation, Depth calibration, Multi-frequency fusion, Point cloud conversion
[31]	3D ToF	ASIC (40 nm)	64 × 32, 50 fps (100 Mhz), 70 mW, Accuracy: 5 cm	In-pixel peak detection, On-chip depth computation, Pixel with embedded histogramming
This Work	3D ToF	FPGA (KU115) 24,657 LUTs, 30,975 FFs, 4 BRAMs, 2 DSPs	1280 × 960, 178 fps (100 Mhz), 1.45 W, MSE ^b^ (HPE) ^c^: 10.03 mm	Dynamic feedback controller (Dynamic area control, Dynamic resolution control), Point-cloud conversion

^a^ Average Precision, ^b^ Mean squared error, ^c^ Hand Pose Estimation

## Data Availability

Dataset available on request from the authors.
